# Mechanisms of Bushenyiqi decoction in the treatment of asthma: an investigation based on network pharmacology with experimental validation

**DOI:** 10.3389/fphar.2024.1361379

**Published:** 2024-03-25

**Authors:** Ziwen Qin, Yujuan Chen, Na Liu, Yonggang Wang, Lili Su, Bin Liang, Chuanjun Huang

**Affiliations:** ^1^ The First Clinical Medical College, Shandong University of Traditional Chinese Medicine, Jinan, Shandong, China; ^2^ Experimental Center, Shandong University of Traditional Chinese Medicine, Jinan, Shandong, China; ^3^ Department of Respiratory, Shandong Provincial Qianfoshan Hospital, Shandong University, Jinan, Shandong, China; ^4^ Department of Respiratory and Critical Care Medicine, Shandong Provincial Hospital Affiliated to Shandong First Medical University, Jinan, Shandong, China

**Keywords:** asthma, Bushenyiqi decoction, network pharmacology, airway inflammation, PI3K/Akt signaling pathway, IL-4, IL-5, IL-13

## Abstract

**Background and purpose:** The Bushenyiqi decoction (BYD), a contemporary prescription of traditional Chinese medicine (TCM), has been observed to significantly ameliorate asthma symptoms in patients based on clinical observations. Although multi-component and multi-target characteristics are important attributes of BYD treatment, its pharmacological effect on asthma and the underlying mechanism of action remain unclear.

**Method:** Network pharmacology: the asthma-related genes were retrieved from the GeneCards and OMIM database. The active constituents of BYD and their corresponding target genes were collected from the TCMSP database. The underlying pathways associated with overlapping targets between BYD and asthma were identified through GO (Gene Ontology) and KEGG (Kyoto Encyclopedia of Genes and Genomes) enrichment analysis. Experimental validation: pulmonary function tests, enzyme-linked immunosorbent assay (ELISA), Hematoxylin and eosin (HE), periodic acid-Schiff (PAS), and Masson’s trichrome stainings were conducted to validate the efficacy of BYD in ameliorating airway inflammation in allergic asthma mice. Western blot (WB) and molecular docking were performed to confirm the involvement of the underlying pathway in BYD treatment of asthma.

**Results:** The results of animal experiments demonstrated that BYD may improve airway responsiveness and suppress airway inflammation in allergic asthma mice. The network pharmacological analysis revealed the involvement of 11 potentially key active components, 9 potential key targets, and the phosphatidylinositol3 kinase-RAC-α serine/threonine-protein kinase (PI3K/AKT) signaling pathway in the mechanism of action of BYD for asthma treatment. Our findings have confirmed that BYD effectively alleviated airway inflammation by targeting interleukin 6 (IL-6), epidermal growth factor receptor (EGFR), and hypoxia inducible factor 1 alpha (HIF1A), with quercetin, kaempferol, and luteolin performing as the pivotal active constituents. BYD may potentially reduce inflammatory cell infiltration in lung tissues by regulating the PI3K/AKT signaling pathway.

**Conclusion:** In conclusion, the integration of network pharmacology and biological experiments has demonstrated that key constituents of BYD, such as quercetin, kaempferol, and luteolin, exhibit targeted effects on IL-6, EGFR, and HIF1A in combating asthma-related inflammation through inhibition of the PI3K/AKT signaling pathway. The findings of this investigation provide evidence supporting the effectiveness of TCM’s “bushenyiqi” therapy in asthma management, as corroborated by contemporary medical technology.

## Introduction

As a significant public health concern associated with a high prevalence of morbidity and mortality, asthma typically presents with clinical symptoms including wheezing, dyspnea, and chest tightness. The condition also presents challenges in terms of treatment efficacy and carries a significant risk of exacerbations, thus posing a substantial global threat to human health and life safety (Reddel et al., 2022). Airway inflammation is central to asthma pathophysiology, which releases various inflammatory mediators associated with airway hyperreactivity (AHR), induces mucous cell metaplasia, smooth muscle proliferation and angiogenesis. Continuous airway remodeling ultimately exacerbates AHR, airway obstruction and airflow restriction in patients with asthma, leading to a progressive decline in lung function (Busse, 1998). T helper (Th) 2-high airway inflammation is commonly observed in individuals suffering from allergic asthma (Fahy, 2015). The activation of Th2 CD4^+^ cells lead to the production of cytokines, such as interleukin (IL) −4, IL-5, and IL-13, which play crucial roles in promoting B-cell class switching towards immunoglobulin E (IgE) synthesis, mast cell differentiation and activation, as well as eosinophilic differentiation and recruitment (Lambrecht et al., 2019). The current therapeutic approaches still face challenges in achieving a cure for asthma, therefore, medical treatment primarily focuses on disease management. The first-line treatment for asthma involves the use of inhaled glucocorticoids (GC), which act as potent anti-inflammatory agents. These medications effectively alleviate asthma symptoms by suppressing immune responses and inhibiting the recruitment and activation of inflammatory cells. However, asthma patients are at a significant risk of experiencing long-term adverse reactions and complications associated with GC use. Furthermore, despite receiving high doses or systemic GC therapy, some patients continue to have uncontrolled asthma due to poor GC sensitivity. Non-compliance with medication and the heavy economic burden are also problems during GC utilization (Djukanovic, 2002; Caramori et al., 2022; Dhar et al., 2023). As a result, there has been a persistent demand for alternative treatments to glucocorticoids among asthma patients (Djukanovic, 2002).

Traditional Chinese medicine (TCM), as an effective complementary and alternative medicine modality characterized by adaptable prescriptions and cost-effectiveness, has progressively garnered attention in Western healthcare. Bushenyiqi decoction (BYD) is a modern herbal formula that was evolved by altering the ingredients of the classic formula “Qiyin decoction” (TCM differentiation records, Chen Shduo, 1687). BYD composes of Epimedii Folium (Yinyanghuo, YYH), Astragalus Membranaceus (Huangqi, HQ), Schisandrae Chinensis Fructus (Beiwuweizi, BWWZ), and Salvia miltiorrhiza (Danshen, DS), with a proportion of 7:10:3:2. Icariin, the main component of Yinyanghuo, has been observed to regulate the imbalance of Th1/Th2 cytokines in asthma mice (Xu et al., 2011), increasing GC receptor expression in the lungs of asthma mice with GC insensitivity (Li et al., 2014). A study found that Danshen extracts exhibit anti-inflammatory effect, effectively attenuating airway inflammation, reducing airway hyperresponsiveness, and preventing airway remodeling in ovalbumin (OVA)-induced asthma mice (Luo et al., 2019). Moreover, salvianolic acid A and tanshinone IIA derived from Danshen can decrease the levels of eosinophils and Th2 cytokines such as IL-4 and IL-13 in allergic asthma mice (Heo and Im, 2019). Schisandra B derived from Beiwuweizi can effectively suppress the expression of Nos2 and Ptgs2, thereby modulating the nuclear factor kappa-B (NF-κB) signaling pathway to play the role of anti-inflammation in asthma mice (Lv et al., 2020). Astragaloside derived from Huangqi may be responsible for the anti-inflammatory effect of Huangqi (Qi et al., 2017). As mentioned above, the available evidence regarding the efficacy of individual herbs in BYD for asthma treatment is limited. However, considering that BYD is a composite formulation comprising four TCM herbs, there remains a need to comprehensively elucidate its essential active constituents and potential mechanisms underlying its therapeutic effects.

The model of network pharmacology proposed by Shao Li has provided a method for exploring the drug-disease interaction from a holistic and systematic perspective, discovering drug targets based on biological networks and clarifying the therapy mechanism of drugs for disease (Chandran et al., 2017). In this study, we employed an integrated approach combining network pharmacology and experimental validation to construct a TCM-disease interaction network for elucidating the key bioactive components, core target genes, and the important pathways of BYD treatment for asthma. The action mechanisms of BYD’s multi-components, multi-targets and multi-links were discussed at the molecular level. The reliability of the network pharmacological analysis results was verified by means of molecular docking and experimental validation, which provided a novel idea for the research and development of asthma drugs based on TCM.

## Materials and methods

### TCMSP and Uniprot database analysis

The TCMSP database (https://old.tcmsp-e.com/tcmsp.php) was queried to retrieve information on each of the four herbs mentioned in BYD, namely, Yinyanghuo, Huangqi, Danshen, and Beiwuweizi. The key pharmacokinetic metrics, namely, oral bioavailability (OB) and drug-likeness (DL), conventionally established as screening criteria with thresholds of greater than 30% and 0.18 respectively. Subsequently, the active components and their corresponding protein target names of the four herbs were obtained. Target names were converted to *Homo sapiens* gene symbols using the Uniprot database (https://www.uniprot.org/), while overlapping target genes and components lacking gene symbols were excluded.

### GeneCards and OMIM database analysis

To identify genes associated with asthma, the keyword “asthma” was utilized to query the GeneCards database (https://www.genecards.org/Search) and OMIM databases (https://www.omim.org/). The genes with a relevance score higher than 1.5 were screened, and any duplicated genes were eliminated. The overlapping genes between BYD target genes and asthma-related genes were identified using the R package “VennDiagram”.

### “Drug-component-target-pathway-disease” network construction

The pathway information pertaining to the overlapping genes of BYD-asthma was acquired from the DAVID database (https://david.ncifcrf.gov/tools.jsp). The table file was prepared and imported into Cytoscape 3.7.2 software, along with comprehensive information on active components of herbs, targets related to these components, and overlapping genes in BYD-asthma. Herbs, active components, genes, pathway IDs, and asthma acted as nodes; the interaction between them acted as edges.

### Protein–protein interactions (PPI) interaction network construction

The STRING database (https://cn.string-db.org/) was utilized to import and identify 75 overlapping targets of BYD-asthma in *Homo sapiens*. The network was then constructed based on a minimum required interaction score of 0.9.

### GO (Gene ontology) and KEGG (Kyoto encyclopedia of genes and genomes) enrichment analysis

Based on the previously published data on overlapping targets between BYD and asthma, the PPI network, and the Bioconductor database (https://bioconductor.org/), the visualization of GO functional enrichment analysis and KEGG pathway enrichment analysis were plotted using the R packages “DOSE”, “clusterProfiler”, and “pathview”.

### Molecular docking

The 3D structures of selected core components were downloaded from the PubChem database (https://pubchem.ncbi.nlm.nih.gov/), and OpenBabel 2.4.2 was utilized to convert the file format of these structures to MOL2. The appropriate 3D protein structures of selected core genes were collected from the RCSB PDB database (https://www.rcsb.org/) and exported as PDB format files. Small molecular and receptor proteins were selected as ligands and receptors, respectively, using AutoDockTools 1.5.7 software optimization. We used AutoDock 4.2.6 software to set docking parameters and then carried out the molecular docking. The binding energy results with values below −1.2 kcal/mol were conventionally regarded as indicative of favorable binding activities. PyMol software performed the docking results visualization.

### Drugs

BYD required Yinyanguho 21g, Huangqi 30g, Wuweizi 9g, and Danshen 6g, which were purchased from Shandong Provincial Hospital of Traditional Chinese Medicine (Jinan, China). Distilled water was added to the above herbs, and the mixture was boiled twice for 30 min each. Subsequently, the resulting liquid was mixed and filtered to remove impurities, yielding 99 mL of BYD with a decocting concentration of 1.5 g/mL.

### Reagents

Aluminum hydroxide [Al(OH)3, alum] was provided by Waltham, USA. OVA, acetyl-β-methyl choline chloride (methacholine), and phosphate-buffered saline (PBS) were purchased from Sigma, United States. Reagents utilized in lung tissue staining (hydrochloric ethanol, masson bluing solution, masson ponceau s staining solution, phosphomolybdic acid solution, aniline blue, paraformaldehyde, xylene, hematoxylin, eosin, periodic acid, and fuchsin basic) were provided by Solarbio Life Sciences (Beijing, China). RIPA buffer, TBST buffer solution, primary antibody dilutions, secondary antibody dilutions, antibody eluent, and goat anti-rabbit IgG labeled with horseradish peroxidase were supplied by Servicebio Biology (Wuhan, China). Antibodies against phosphatidylinositol3 kinase (PI3K, cat.4691T), phospho-PI3K (P-PI3K, cat.17366S), RAC-α serine/threonine-protein kinase (AKT, cat.4691T), phospho-AKT (P-AKT, cat.4060T), and glyceraldehyde-3-phosphate dehydrogenase (GAPDH, cat. 2118T) were purchased from Cell Signal Technology (Beverly, MA, United States).

### Animals

A total of 24 pathogen-free female BALB/c mice (aged 7–9 weeks, weight 15 ± 2 g) were purchased from the Experimental Center of Shandong University (Jinan, China). The mice were housed in a pathogen-free standard environment for 1 week, during which they received adjustable feeding. The environmental conditions included a 12-h light/dark cycle, a temperature of 20°C, and a relative humidity of 60%. All animal experiments are performed in accordance with the regulations and guidelines from the National Institutes of Health (NIH) Guide for the Care and Use of Laboratory Animals (National Research Council Committee for the Update of the Guide for the and Use of Laboratory, 2011). All animal experimental designs and procedures were approved by the ethics committee on animal care of Shandong Provincial Hospital & Shandong First Medical University (Jinan, China).

### Allergic asthma model

**FIGURE 1:**
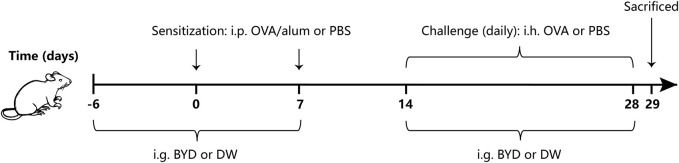
Protocol of OVA-induced allergic asthma and administration of BYD. Mice were sensitized on days 0 and 7, following by daily OVA challenges on days 14–28. BYD treatment group was treated i.g. for 14 days prior to the second sensitization, and for 14 days next to the first OVA inhalation challenge. BYD, bushenyiqi decoction; OVA, ovalbumin; PBS, phosphate-buffered saline; alum, aluminum hydroxide; DW, distilled water; i.p., intraperitoneal injection; i.g., intragastric administration; i.h., atomizing inhalation.

We determined the method for asthma modeling based on slight improvements of the methods in the previous studies (Huang et al., 2021; Huang et al., 2022), while the timing and concentration of administration were determined by preliminary experiment of this study. 24 mice began to enter the asthma modeling stage after 7 days of adaptive feeding. The mice were randomly divided into three groups with eight mice in each group: normal control group (control), OVA-induced asthma group (model), BYD treatment group (BYD). In asthma groups, mice were sensitized by intraperitoneal injection (i.p.) of a mixed solution consisting of 20 µg OVA, 2 mg alum on day 0 and day 7. From day 14–28, mice were challenged by atomizing inhalation (i.h.) of an OVA solution consisting of 3% OVA for 30 min each day. The sensitizing and challenging materials in control group were replaced by an equal dose of PBS. 14 days before the second sensitization, BYD (40 g/kg) were given once a day by intragastric administration (i.g.) in BYD treatment group, while the same dose of distilled water was given to control group. From day 14–28, 1 h before each challenge, the treatment measures and doses for each group were the same as above. 24 h after the last challenge, lung function was tested, bronchoalveolar lavage fluid (BALF), blood, and lung tissues were taken (Figure 1).

### Airway responsiveness measurement

The measurement of airway responsiveness was conducted invasively using the PFT pulmonary maneuvers system (Buxco Electronics, Inc., Troy, NY, United States). On day 29, mice were anesthetized with pentobarbital (50 mg/kg) and subsequently underwent tracheotomy, followed by attachment of the trachea to the pulmonary function test system. Initially, atomization was performed using a PBS solution, followed by the addition of methacholine at concentrations of 3.125, 6.25, and 12.5 mg/mL for subsequent atomization with an interval of 5 min each. After 10s of atomization, pulmonary resistance and pulmonary compliance were recorded. The ratio of lung resistance (RL) and dynamic lung compliance (Cdyn) to the baseline (PBS challenge) level was employed as an indicator for assessing airway responsiveness of mice.

### BALF collection and inflammatory cell analysis

After assessing airway responsiveness, the mice were euthanized and the trachea was carefully dissected. A tracheal catheter was inserted and 1 mL of PBS was used for triple irrigation. The collected BALF was centrifuged at 4°C and 1200 R/min for 5 min, and the supernatant was absorbed and stored in the refrigerator at −80°C. The precipitated cells were re-suspended with 1 mL of PBS. The total number of cells and the number of inflammatory cells in BALF were measured by the IDEXX ProCyte DX blood analyzer (IDEXX Laboratories Inc., Westbrook, ME, United States).

### Enzyme-linked immunosorbent assay (ELISA)

Th2 cytokine expressions in BALF and serum of mice were tested using ELISA to evaluate the airway inflammation in asthma mice. Fresh blood from mice was left at room temperature for 1 h, then centrifuged at 4°C and 2500R/min for 10 min, reserving the supernatant for detection. The BALF supernatant was removed from the −80°C refrigerator and dissolved over ice cubes. The levels of IL-4, IL-5, and IL-13 in serum and BALF were respectively assessed using the ELISA kit (R&D Systems, Inc.) following the standard procedures described in the product instructions.

### Hematoxylin and eosin (HE), periodic acid-Schiff (PAS), and Masson’s trichrome staining

Fresh mice lung tissues were fixed in 4% paraformaldehyde for 24 h, followed by dehydration using a gradient concentration of alcohol and immersion in a wax solution. Subsequently, the tissues were embedded and sliced into sections of 4–5 μm thickness for HE, PAS, and Masson’s trichrome staining.

HE staining: Paraffin sections were dewaxed using xylene, followed by hydration in a gradient concentration of alcohol and subsequent washing with distilled water. The sections were stained with hematoxylin for a duration of 10 min, followed by differentiation in 1% hydrochloric ethanol for 30 s. Subsequently, the sections were stained with 0.5% eosin for a period of 1 min, and rinsed with distilled water after each step. Finally, dehydrated the sections in gradient concentration alcohol, transparentized with xylene, then sealed using neutral gum.

PAS staining: The sections were dewaxed and hydrated using the same aforementioned steps, followed by impregnation in a 5% periodate alcohol solution for 8 min and subsequent washing in 70% ethanol. Subsequently, the sections were immersed in a colorless fuchsin basic solution for 20 min, rinsed, and finally stained with hematoxylin for 30 s to visualize cellular structures. Dehydrated and transparentized sections as above.

Masson’s trichrome staining: Dewaxing and hydrating the sections as above. Stained cell nucleus with hematoxylin for 3 min, dripped acidic alcohol solution for 5 s for differentiating, added masson bluing solution for 5 min to reverse blue, while rinsing in distilled water after each step. Stained with masson ponceau s staining solution for 5 min, and drip-washed with weak acid working liquid. Differentiated with 1% phosphomolybdic acid solution for 2 min and drip-washed with weak acid working liquid. Stained with aniline blue for 2 min, then dehydrated and transparentized sections as above.

HE staining was used to evaluate the airway inflammation. The inflammation surrounding the bronchus and blood vessels was observed using an optical microscope in each section, with the inflammation score ranging from 0 to 5 points (Zhang et al., 2023). PAS staining was used to assess airway mucus secretion, while masson’s trichrome staining evaluated collagen deposition. The positive-staining airway wall area (Wat) and bronchial basal membrane circumference (Pbm) were quantified using Image J software. The results of PAS and Masson’s trichrome staining were presented as the ratio of Wat to Pbm.

### Western blot analysis

Add protein lysate on ice to dissolve lung tissues for 30 min. Tested the protein concentration according to the instructions of the BCA protein quantification assay kit (G2026, Servicebio Biology, Wuhan, China). After the electrophoresis, the separation glue was immersed in the membrane transfer solution configured using 10% PAGE color (red) gel ultra-fast formulation kit (G2043-50T, Servicebio Biology, Wuhan, China) and transferred to the PVDF membrane for 30 min at a constant current of 300 mA. At room temperature, imprint with 5% skim milk in TBST buffer for 2 h, and then incubate overnight at 4°C with the following primary antibodies: anti-P-PI3K (1:500), anti-PI3K (1:1,000), anti-AKT (1:1,000), anti-P-AKT (1:1,000), anti-GAPDH at 1:5,000). On the second day, washed with TBST for 3 times, then diluted the second antibody (goat anti-rabbit IgG labeled with horseradish peroxidase) with TBST at a ratio of 1:3,000, incubated in a shaker at room temperature for 1 h. Reapply the mixed ECL luminescent liquid onto the PVDF membrane and initiate ECL chemiluminescence by employing the ECL chemiluminescence kit (G2014-50ML, Servicebio Biology, Wuhan, China). Integrated densities were measured by AIWBwell analysis software, and the ratio of indicator integrated density to parameter integrated density (relative content of the sample) was considered as the evaluation index.

### Statistical analysis

The obtained data are presented as a mean ± standard error of the mean (SEM). The systematic analysis was performed with IBM SPSS Statistics 25 software using one-way ANOVA comparing data between multiple groups which were normally distributed. A value of *p* < 0.05 was considered to have statistically significant differences between groups.

## Results

### Collection of BYD active components and BYD-asthma overlapping targets

**FIGURE 2:**
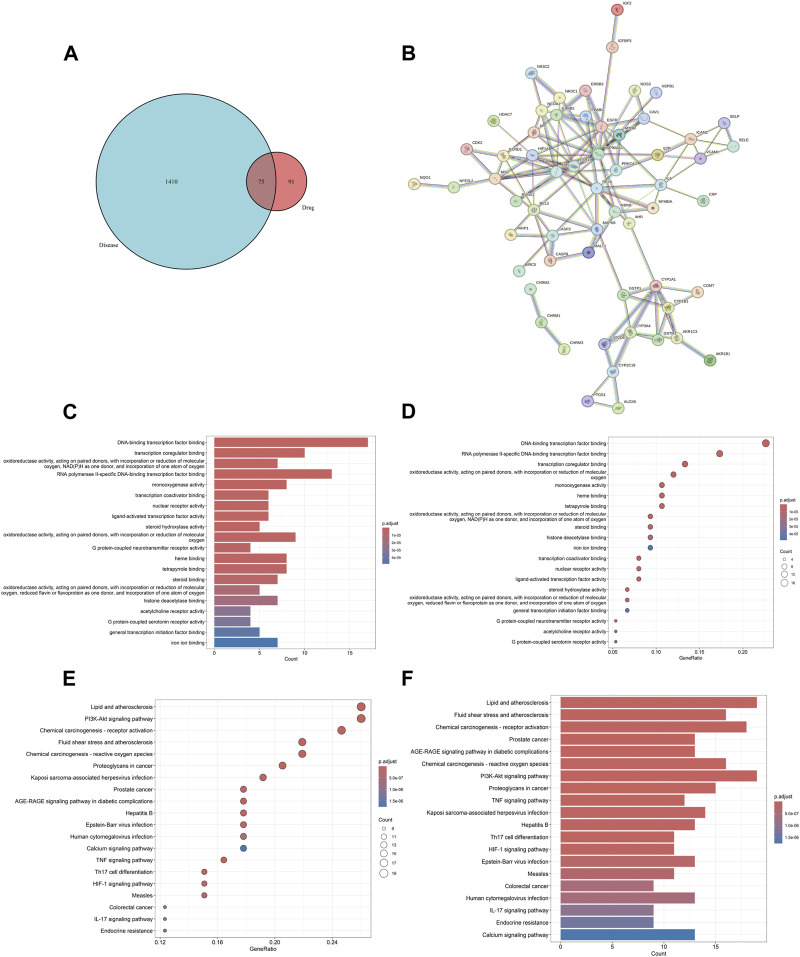
BYD-asthma overlapping targets identification, PPI network, and GO/KEGG enrichment analysis. **(A)** The overlap in BYD targets in TCMSP database and differential genes of asthma from Genecards and OMIM datasets. **(B)** The analysis of the PPI network and clusters. **(C)** Barplot image from GO functional enrichment analysis of 75 protein targets of BYD in asthma. **(D)** Dotplot image from GO analysis. **(E)** Dotplot image from KEGG pathway enrichment analysis. **(F)** Barplot image from KEGG analysis.

The screening criteria in the TCMSP database were set as OB ≥ 30% and DL ≥ 0.18 to search for the four drugs, resulting in the identification of 116 active components and their corresponding target protein names. The protein names were mapped to 926 gene symbols of *Homo sapiens* using the Uniprot database. After excluding 767 duplicate genes and 11 active components lacking gene symbols, we identified 166 BYD targets, 21 YYH components, 18 HQ components, 6 BWWZ components, and 60 DS components. We identified 1,485 asthma-related genes through screening in the GeneCards database, eliminating 39 duplicate genes obtained from the OMIM database. The identification of the 75 DBY-asthma overlapping genes and the visualization image were yielded via running the R package “VennDiagram” (Figure 2A).

### Drug-component-target-pathway-disease network and key component analysis

**FIGURE 3:**
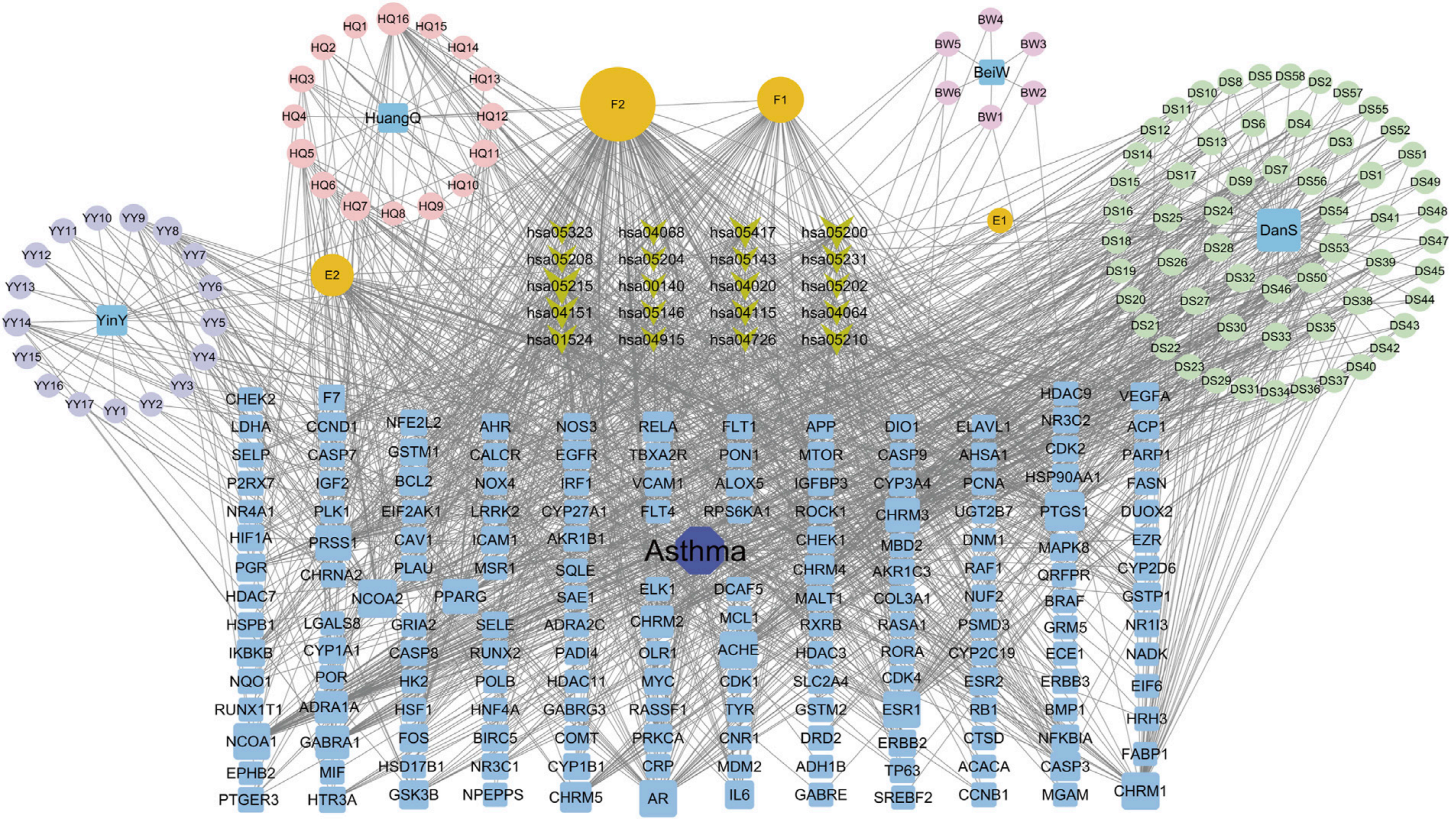
The “Drug-component-target-pathway-disease” network for BYD active components, 75 BYD-asthma overlapping targets, and the signaling pathways involved with the targets.

The interaction network was constructed utilizing the Cytoscape software (Figure 3). The engagement information of the active components in the network was acquired through utilization of a network analysis tool. When degree, betweenness centrality, and closeness centrality were considered as the indicators, 11 potentially key active components in the BYD treatment for asthma were identified (Table 1): quercetin (MOL000098), kaempferol (MOL000422), luteolin (MOL000006), isorhamnetin (MOL000354), tanshinone iia (MOL007154), anhydroicaritin (MOL004373), 7-O-methylisomucronulatol (MOL000378), dan-shexinkum d (MOL007093), NSC 1224219 (MOL007149), α-amyrin (MOL006824), and salviolone (MOL007145).

**TABLE 1:** The first 11 potential active components from “Drug-component-target-pathway-disease” network analysis.

Mol ID	Mol name	ID	Degree	Betweenness centrality	Closeness centrality
MOL000098	quercetin	F2	156	0.21422652	0.48178808
MOL000422	kaempferol	F1	69	0.04786254	0.41930836
MOL000006	luteolin	E2	58	0.06480857	0.41335227
MOL000354	isorhamnetin	HQ5	18	0.02245011	0.39754098
MOL007154	tanshinone iia	DS50	22	0.03304271	0.38390501
MOL004373	Anhydroicaritin	YY8	17	0.01068861	0.38089005
MOL000378	7-O-methylisomucronulatol	HQ7	19	0.01367089	0.37890625
MOL007093	dan-shexinkum d	DS24	16	0.00972813	0.37792208
MOL007149	NSC 122421	DS53	18	0.05787388	0.375
MOL006824	α-amyrin	DS54	14	0.03734119	0.36557789
MOL007145	salviolone	DS46	15	0.01252574	0.35837438

### PPI network construction and key target identification

The PPI network, consisting of 75 nodes and 126 edges, was constructed by importing 75 BYD-asthma overlapping targets into the STRING database (Figure 2B). And the network analysis was performed by Cytoscape software. After deleting targets lower than the mean value of degree (4.271186441), betweenness centrality (0.057581465), and closeness centrality (0.338852949), the remaining 9 targets were considered as the potential key targets in BYD treatment for asthma (Table 2): heat shock protein 90 alpha family class A member 1 (HSP90AA1), estrogen receptor 1 (ESR1), proto-oncogenec-Fos (FOS), epidermal growth factor receptor (EGFR), mitogen-activated protein kinase 8 (MAPK8), B-cell lymphoma-2 (BCL2), proto-oncogene Myc (MYC), IL6, caspase 3 (CASP3), and most of which are involved in the PI3K/AKT signaling pathways (Laboratories, 2024).

**TABLE 2:** The nine potential target genes obtained from PPI network analysis.

Gene name	Degree	Betweenness centrality	Closeness centrality
HSP90AA1	16	0.27858248	0.47008547
ESR1	14	0.1670525	0.45454545
FOS	10	0.06756222	0.40145985
EGFR	10	0.10898703	0.39285714
MAPK8	8	0.21672917	0.42635659
BCL2	8	0.07690206	0.39285714
MYC	8	0.09662755	0.37414966
IL6	7	0.10976059	0.36912752
CASP3	6	0.05834265	0.3525641

### Functional analysis and gene pathways

GO and KEGG enrichment analysis can facilitate a comprehensive understanding of the underlying molecular mechanisms involved in BYD treatment for asthma, enabling the identification of functional biases and significant pathways throughout its therapeutic process. The barplot and dotplot images of GO enrichment analysis (Figures 2C, D) implied that BYD might exert its therapeutic effect via regulating DNA-binding transcription factor activity and promoting transcription coregulator binding to stimulate the transcription of anti-inflammatory molecules, thereby regulating the body’ state of oxidative stress. Visual images of KEGG pathway enrichment analysis (Figures 2E, F) indicated the BYD-asthma targets were mainly concentrated in the PI3K/AKT signaling, tumor necrosis factor (TNF) signaling, Th17 cell differentiation, hypoxia inducible factor-1 (HIF-1) signaling, and IL-17 signaling pathways, which are closely associated to the immune inflammation in asthma. According to the identified key genes predominantly involved in the PI3K/AKT signaling pathway and the results of KEGG analysis, it can be inferred that the PI3K/AKT signaling pathway plays a pivotal role in BYD’s mechanisms of action for asthma treatment.

### The interaction between BYD and targets

**FIGURE 4:**
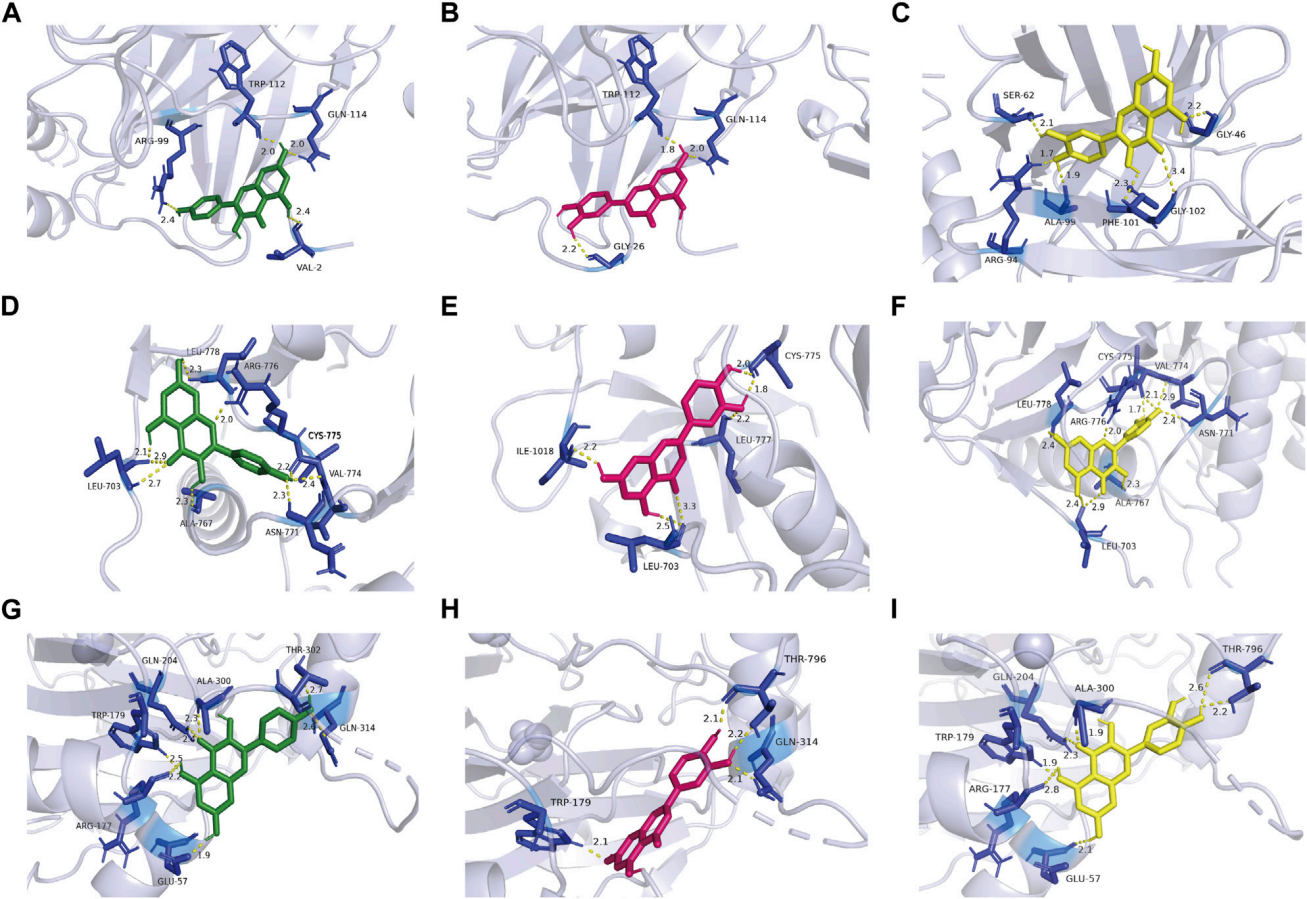
Kaempferol, luteolin, and quercetin docking models with IL-6 (4zs7), EGFR (3ug2), and HIF1A (1h2k), respectively. The length was added around the dashed lines that represented the hydrogen bonds. **(A)** IL-6 action model with kaempferol. **(B)** IL-6 action model with luteolin. **(C)** IL-6 action model with quercetin. **(D)** EGFR action model with kaempferol. **(E)** EGFR action model with luteolin. **(F)** EGFR action model with quercetin. **(G)** HIF1A action model with kaempferol. **(H)** HIF1A EGFR action model with luteolin. **(I)** HIF1A action model with quercetin.

Through literature search, we found that the research on IL6, EGFR, and HIF1A to asthma was relatively richer compared with these of other targets. Moreover, we selected them as core target genes based on their significance in both the PPI network and the PI3K/AKT signaling pathway (Figure 4). We selected the top three active components out of the 11 key components, namely, quercetin, kaempferol, and luteolin, based on their extensive literature support and high centrality in the drug-component-target-pathway-disease network. Subsequently, Autodock software was employed to dock the 3 core targets with the 3 core components. The results of nine dockings showed binding energies ranging from −5.26 to −6.76 kcal/mol, which were all much lower than −1.2 kcal/mol (Table 3). The highest binding activity was observed between IL-6 and luteolin, with a binding energy of −6.13 kcal/mol. Similarly, quercetin exhibited the highest binding activity towards EGFR, with a binding energy of −6.76 kcal/mol. Additionally, HIF1A showed the strongest affinity for kaempferol, with a binding energy of −5.41 kcal/mol. Our molecular docking results demonstrated that the three core active components of BYD can directly target the three core targets of asthma, implying that quercetin, kaempferol, and luteolin in BYD may exert therapeutic effects by intervening and modulating the PI3K/AKT signaling pathway related to asthma.

**TABLE 3:** Binding energy of 9 molecular dockings [kaempferol, luteolin, and quercetin docking with IL-6 (4zs7), EGFR (3ug2), and HIF1A (1h2k), respectively].

Target	Active component	Binding energy (kcal/mol)
IL6	quercetin	−5.47
	kaempferol	−5.92
	luteolin	−6.13
EGFR	quercetin	−6.76
	kaempferol	−6.38
	luteolin	−5.99
HIF1A	quercetin	−5.35
	kaempferol	−5.41
	luteolin	−5.26

### BYD alleviated AHR to acetylcholine chloride in mice models of allergic asthma

**FIGURE 5:**
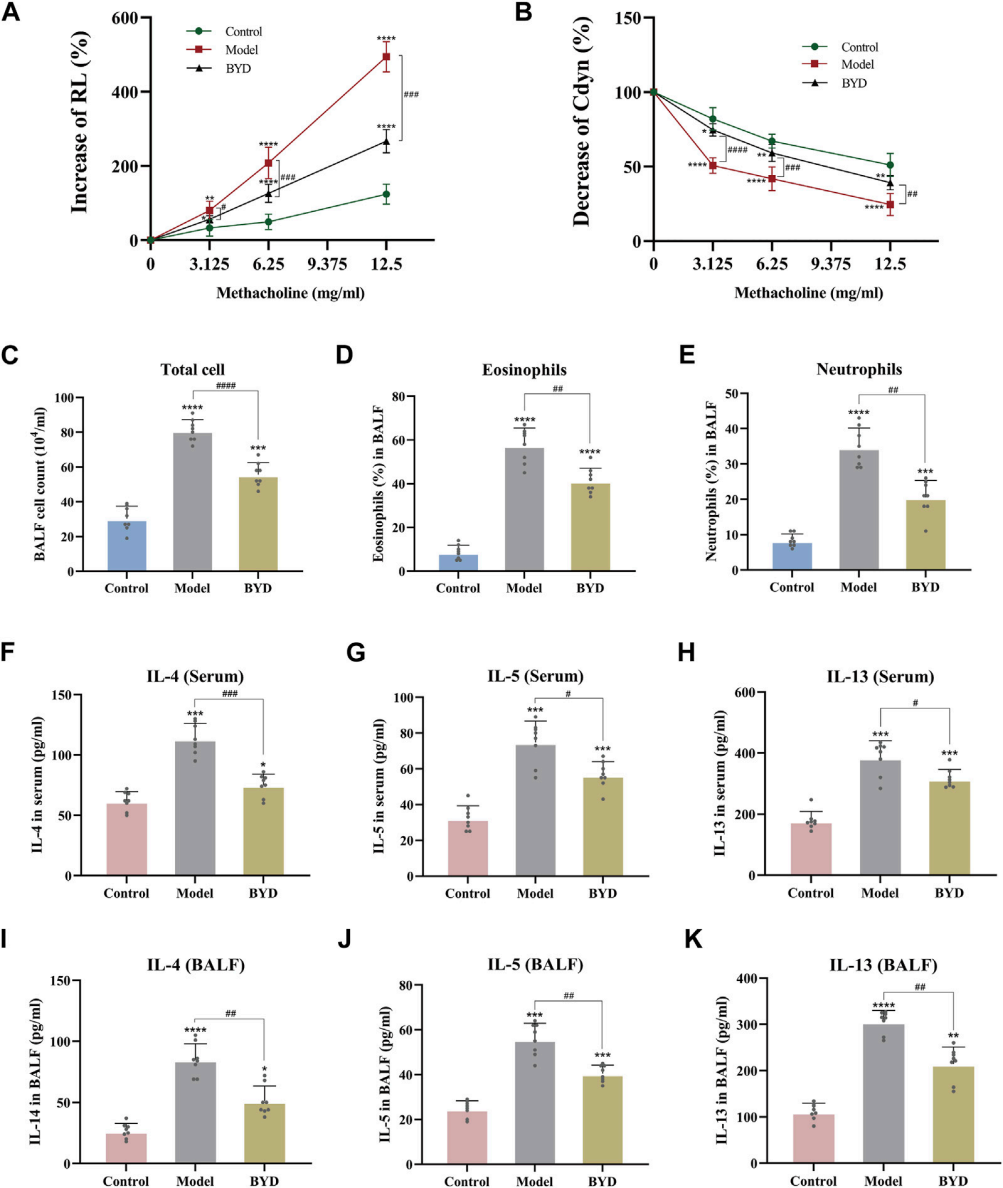
BYD alleviated the airway inflammation and airway hyper-responsiveness in mice models of allergic asthma. Airway responsiveness of three groups of mice was implied by measuring RL **(A)** and Cydn **(B)** in response to increasing concentrations of methacholine. Inflammatory cell analysis in BALF including total cell count **(C)**, eosinophil ratio **(D)**, and neutrophil ratio **(E)**. Th2 cytokines expression including IL-4, IL-5, IL-13 in serum **(F–H)** and BALF **(I–K)** of mice were tested using ELISA to evaluate the airway inflammation. Data were expressed as mean ± SEM (*n = 8*). *p < 0.05, **p < 0.01, ***p < 0.001, and ****p < 0.0001 compared to control group. ^#^ *p* < 0.05, ^##^ *p* < 0.01, ^###^ *p* < 0.001, and ^####^ *p* < 0.0001 BYD group compared to model group. RL, lung resistance; Cdyn, dynamic lung compliance; IL-4, interleukin 4; IL-5, interleukin 5; IL-13, interleukin 13; BALF, bronchoalveolar lavage fluid; BYD, bushenyiqi decoction.

The expressions of RL and Cydn in 3 groups of mice were measured under different concentrations of methacholine by the pulmonary maneuvers system, evaluating the changes compared with the baseline. As shown in (Figure 5A), with the increase in the concentration of methacholine used for challenge, the increase level and rising trend were most significant in the model group compared with control group. BYD could significantly reduce the RL of asthma mice in the face of methacholine stimulation. The Cydn displayed a decreasing trend when the concentration of methacholine increased in 3 groups (Figure 5B). BYD performed a significant effect in resisting the decreasing tendency of Cdyn. In the RL and Cydn line graphs, the line trend and expression level of BYD group were most similar to those of the control group, indicating that the BYD treatment could effectively alleviate AHR in mice with allergic asthma.

### BYD decreased the levels of inflammation cells and cytokines expression in BALF and serum of asthma mice

By assessing the inflammatory cells in BALF of mice, including total cells, eosinophile percentage and neutrophil percentage, it was found that the inflammation degree of mice in model group was significantly elevated than that in control group, while BYD treatment could significantly reduce the levels of total inflammatory cells, eosinophils and neutrophils in BALF of asthma mice (Figures 5C–E). The levels of Th2 inflammatory cytokines, including IL-4, IL-5, and IL-13, were quantified in both BALF and serum samples from mice via ELISA (Figures 5F–K). Results displayed that BYD could significantly reduce the level of inflammatory cytokines in asthma mice, indicating the important regulatory and inhibitory effects of BYD on airway inflammation in asthma mice.

### BYD alleviated inflammation and pathological changes in lung tissues of asthmatic mice

**FIGURE 6:**
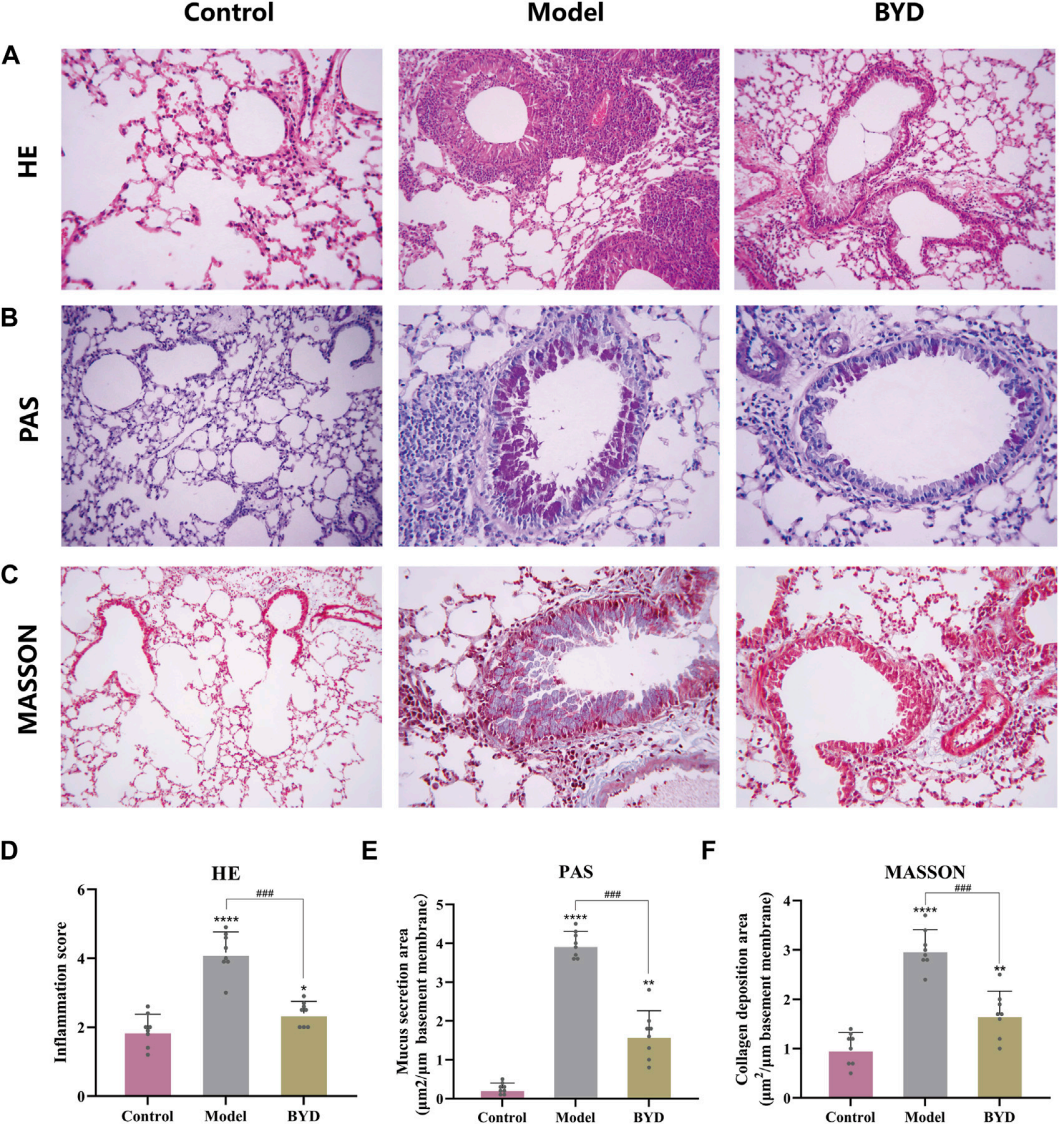
BYD alleviates airway inflammation and airway remodeling in mice models of allergic asthma. HE staining of lung tissues **(A)** and inflammation scores around the trachea and blood vessels **(D)**. PAS staining of lung tissue **(B)** and the quantitative analysis of airway mucus secretion **(E)**. Masson staining of lung tissue **(C)** and the quantitative analysis of airway collagen deposition **(F)**. Data are expressed as mean ± SEM (*n = 8*). *p < 0.05, **p < 0.01, and ****p < 0.001 compared to control group. ^###^ *p* < 0.001 BYD group compared to model group. HE, hematoxylin and eosin; PAS, periodic acid-schiff; BYD, bushenyiqi decoction.

The HE staining technique was employed to examine the extent of inflammatory infiltration surrounding the trachea and blood vessels in lung tissues of asthmatic mice. As shown in (Figure 6A), the bronchus in lung tissues of control group mice exhibited a normal morphology, with uniform thickness of the tube wall, unobstructed lumens, and clear delineation of alveoli. In contrast, asthmatic mice displayed thickened tube walls, deformed lumens, damaged alveoli and significant infiltration of high-density inflammatory cells around the tube wall and blood vessels. Asthmatic mice with BYD treatment showed significantly alleviated inflammatory infiltration and reduced inflammatory cell number. The trachea walls exhibited a decrease in thickness, and there was an improvement in the shape of alveolar walls. Additionally, a significant reduction in the inflammatory score was observed compared to the model group (Figure 6D). Additionally, PAS staining and Masson’s trichrome staining were employed to further examine collagen deposition and mucus secretion in lung tissues. It was observed that BYD treatment led to a significant reduction in collagen deposition along the endotracheal lining and mucous secretion within the lumen (Figures 6B, C, E, F), indicating notable improvements compared to the model group. Therefore, we considered that BYD may play an important role in reducing airway inflammation, collagen deposition, and mucus secretion in asthma.

### BYD reduced the phosphorylation of PI3K and AKT in lung tissues of asthma mice model

The network pharmacology results mentioned above revealed that the PI3K/AKT signaling pathway may potentially contribute to the therapeutic effects of BYD. In order to further validate the involvement of this pathway in BYD-mediated suppression of airway inflammation in asthmatic mice, we used Western blot analysis to determine the protein expression of PI3K, phosphorylated PI3K, AKT, and phosphorylated AKT in lung tissues, with GAPDH serving as an internal control.

**FIGURE 7:**
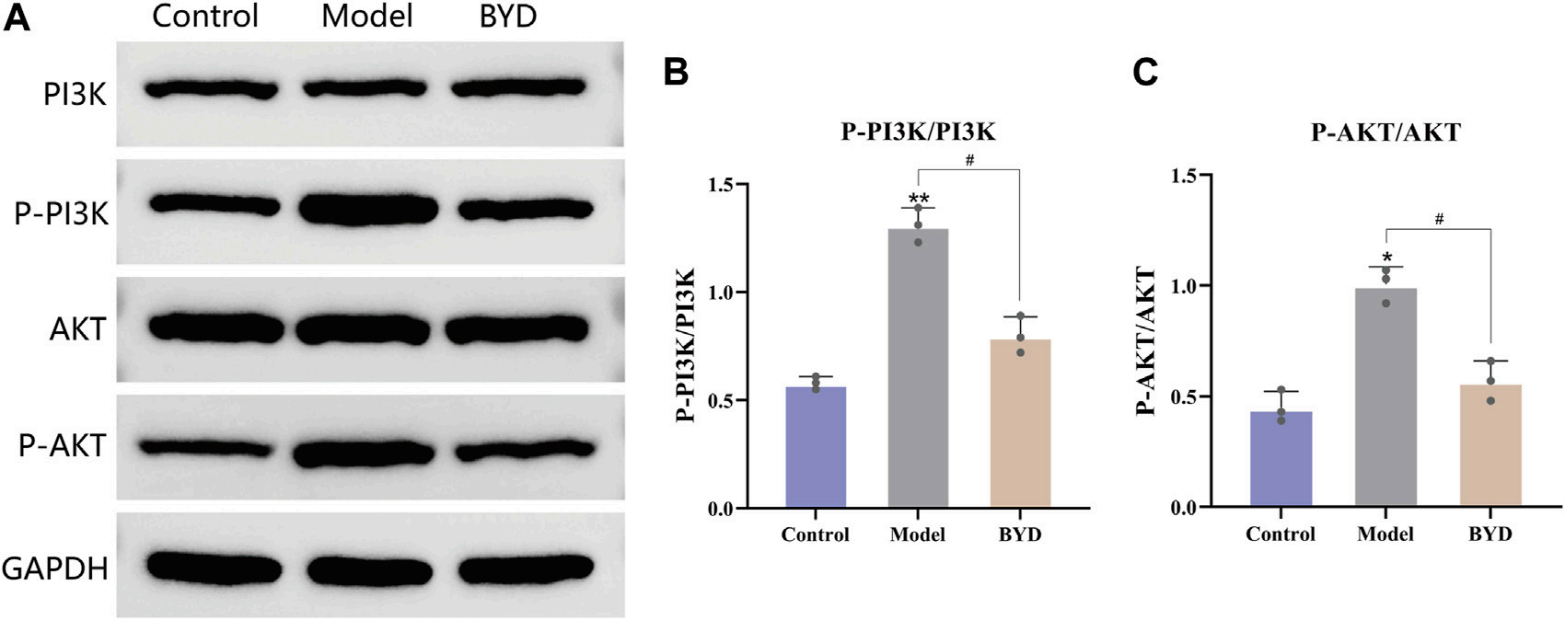
BYD downregulated the PI3K-Akt pathways in mice model of allergic asthma. **(A)** The protein expression levels of p-PI3K, PI3K, p-AKT, AKT in mice lung tissues were measured by Western blot analysis. **(B)** The ratios of p-PI3K and PI3K protein expression in three groups. **(C)** The ratios of p-AKT and AKT protein expression in three groups. Data are expressed as the mean ± SEM (*n = 3*). *p < 0.05 and **p < 0.01 compared to control group. ^#^ *p* < 0.05 BYD group compared to model group. P-, phosphorylated; PI3K, Phosphatidylinositol-3-kinase; AKT, Protein kinase B; GAPDH, glyceraldehyde-3-phosphate dehydrogenase; BYD, bushenyiqi decoction.

In order to better ascertain the influence of BYD to asthmatic mice, we take the left lower lobe of lung tissues from each group of mice and conduct extractions of tissue homogenate and proteins, then repeated the WB detection for three times. The remaining lung tissues were used for conduct different experiments, such as slicing and staining of lung tissues. The expression levels of phosphorylated PI3K and AKT were significantly increased in the model group, suggesting a close association between airway inflammation in asthmatic mice and activation of the PI3K/AKT signaling pathway (Figure 7A). The protein expressions of phosphorylated PI3K and AKT in lung tissues of BYD-treated mice were significantly diminished compared to those in the model group, implying that BYD may modulate airway inflammation in asthma mice by suppressing the phosphorylation of PI3K and AKT and inhibiting the activation of the PI3K/AKT signaling pathway (Figures 7B, C).

## Discussion

Bronchial asthma is a heterogeneous disease characterized by complex and variable pathogenesis, with airway inflammation being considered the fundamental pathological manifestation (Sockrider and Fussner, 2020). The immune response exhibits a Th2 cell bias, while the involvement of inflammatory cells such as eosinophils and neutrophils can drive asthmatic airway inflammation (Fahy, 2015). The expression of inducible nitric oxide synthase (iNOS) in airway epithelial cells shows an increase driven by Th2-high immune response, thereby catalyzing the production of a large amount of nitric oxide (NO) from L-arginine. Therefore, fraction of exhaled NO (FeNO), as a biomarker, can reflect airway inflammation and hyperresponsiveness, with high sensitivity and specificity. Therefore, FeNO measurement is of great significance in the early diagnosis, treatment, and postoperative detection of asthma (Karampitsakos and Gourgoulianis, 2016; Karampitsakos et al., 2018). In recent years, extensive research has focused on elucidating the interplay between the activated PI3K/AKT signaling pathway and asthma-related airway inflammation and remodeling (Rao et al., 2017; Athari, 2019). It is believed that the pathway involves in the activation of Th2 cells and the infiltration of inflammatory cells which promote airway inflammation and remodeling (Karampitsakos et al., 2019). Therefore, drugs targeting the PI3K/AKT signaling pathway may play a potentially positive role in regulating airway inflammation and remodeling. Multiple Chinese medicine prescriptions have been developed following the traditional Chinese medicine therapy method of “bushenyiqi”. Among them, BSYQF demonstrates the ability to modulate the balance between Th1 and Th2-Th17 immune responses, thereby reducing asthma attacks induced by respiratory syncytial virus (RSV) (Wang et al., 2014). In addition, M-BYF was also found to alleviate AHR, airway inflammation and remodeling in asthma mice by negatively regulating the vasoactive intestinal peptide-vasoactive intestinal peptide receptor type 2 (VIP-VPAC2) signaling pathway (Huang et al., 2021). The representative herbs of “bushenyiqi” therapy method, namely, Yinyanghuo and Huangqi, along with their monomer components, have been demonstrated to possess inhibitory effects on airway inflammation in asthma (Yi et al., 2020; Bi et al., 2022). However, the pharmacology and mechanism of modern prescription BYD, guided by the concept of “bushenyiqi” and emphasizing the significance of Yinyanghuo and Huangqi as key constituents, remain to be elucidated. Therefore, this study tried to explore the main biological active components and their molecular mechanisms in BYD therapy for asthma through the integration of network pharmacology and experimental validation.

The presence of airway inflammation is widely recognized as a crucial determinant reflecting the heterogeneity of asthma, while also serving as a fundamental condition underlying AHR, mucus hypersecretion, and airway remodeling (Banno et al., 2020). Repressing airway inflammation is of paramount importance in ameliorating asthma symptoms, diminishing the frequency of attacks, and retarding disease progression. Our experimental study found that asthma mice treated with BYD were significantly improved in airway responsiveness. Total count of inflammatory cells and the ratio of eosinophils and neutrophils in BALF of BYD-treated mice decreased significantly, as well as the levels of Th2 inflammatory cytokines in serum and BALF. In addition, the lung tissue staining revealed that BYD could obviously reduce inflammatory cell infiltration, mucus secretion and collagen deposition in lung tissues of asthma mice, confirming the significant inhibitory effect of BYD on airway inflammation in asthmatic mice.

In order to further investigate the key constituents of BYD involved in asthma treatment and the gene targets of BYD treatment, we collected active components that regulate genes associated with asthma and identified overlapping gene targets between BYD and asthma. Subsequently, molecular docking was conducted to validate their binding activities. Network pharmacological research found that 4 herbs in BYD had 105 active components and 166 target genes. 75 target genes of BYD were obtained when they were intersected with asthma-related genes. By constructing the BYD-asthma interaction network, we identified 11 active components that exhibit potential efficacy in BYD treatment of asthma. Notably, quercetin, luteolin and kaempferol demonstrated robust interactions with target genes. Quercetin can inhibit the phosphorylation of AKT, suppress the production of inflammatory mediators and pro-inflammatory cytokines, thus improving Th1/Th2 balance and oxidative stress, exerting a certain anti-allergic and anti-oxidant effect. In addition, quercetin exhibits potential therapeutic effects on airway inflammation in neutrophilic asthma by attenuating neutrophil-mediated airway inflammation through modulation of M1 macrophage polarization (Moon et al., 2008; Park et al., 2009; Ganesan et al., 2012; Mlcek et al., 2016). Kaempferol can inhibit airway inflammation and airway remodeling by interfering with signal transduction in mast cells and recombinant nicotinamide adenine dinucleotide phosphate oxidase 4 (NOX4)-mediated autophagy, effectively improving allergic asthma symptoms in guinea pigs (Shin et al., 2015; Molitorisova et al., 2021; Xu et al., 2023). Moreover, it can effectively suppress zearalenone-induced oxidative stress and apoptosis by modulating the PI3K/AKT-mediated nuclear factor erythroid-2-related factor 2 (Nrf2) signaling pathway (Rajendran et al., 2020). Luteolin exhibited inhibitory effects on airway inflammation in mice with allergic or neutrophilic asthma, and it modulated the PI3K/AKT/mammalian target of rapamycin (mTOR) signaling pathway to inhibit autophagy in allergic asthma (Wang et al., 2021; Qiao et al., 2023). The above studies suggested that asthma may be improved by inhibiting various of pathways involved in the inflammatory response. And the 3 key components of BYD all have significant anti-inflammatory effects related to the modulation of the PI3K/AKT signaling pathway. The PI3K/AKT signaling pathway is involved in multiple biological processes such as Th2 cell differentiation, antigen recognition and presentation by toll-like receptor 2 (TLR2), NF-κB activation by reactive oxygen species (ROS), and mTOR phosphorylation, consequently, it exerts an impact on asthma inflammation and airway epithelial repair process (Athari, 2019). Inhibition of the PI3K/AKT signaling pathway and its downstream biological processes may exert a potential therapeutic effect for alleviating airway inflammation in asthma.

Through the construction of PPI interaction network, it was found that HSP90AA1, ESR1, FOS, EGFR, MAPK8, BCL2, MYC, IL6 and CASP3 targeted by BYD active components were mainly involved in the treatment process of asthma. Importantly, all these protein genes were found to be involved in the PI3K/AKT signaling pathway. GO enrichment analysis implied that BYD may regulate DNA-binding transcription factor activity and promoting transcription coregulator binding to stimulate the transcription of anti-inflammatory molecules, thus exerting its therapeutic effect. Moreover, KEGG pathway analysis indicated the PI3K/AKT signaling pathway may play an important role in BYD’s mechanism of action, which are closely associated to the secretion of various (anti-)inflammatory cytokines, oxidative stress status, and immunoregulation in asthma, which strongly support the notion that the PI3K/AKT signaling pathway may hold significant relevance in BYD treatment for asthma. The 3 key active components were molecule-docked with the 3 key genes, resulting in all good binding activities from 9 times of docking, implying that BYD may regulate asthma airway inflammation by interfering in the PI3K/AKT signaling pathway. The protein expressions of the PI3K/AKT signaling pathway were measured by western bolt, calculating the ratio of phosphorylated PI3K and AKT to PI3K and AKT. Results showed that the PI3K/AKT signaling pathway was activated in lung tissues of asthma mice, while the pathway was significantly inhibited in asthma mice treated with BYD. It was speculated that BYD may inhibit the activation of the PI3K/AKT signaling pathway and its downstream inflammatory mediators by blocking the phosphorylation process of PI3K and AKT, thereby reducing airway inflammation. Through integrated network pharmacology and experimental validation, it was found that BYD’s mechanism for treating asthma is characterized by multiple targets and multiple pathways. The primary targets are predominantly localized within the PI3K/AKT signaling pathway. By down-regulating the phosphorylation of PI3K and AKT, BYD may inhibit the expression of inflammatory factors and inflammatory cells induced by the activation of downstream pathways, thus alleviating airway inflammation in allergic asthma mice, implying the potential effect of BYD in the treatment of asthma. However, how BYD act on airway inflammation in asthma through downregulation the PI3K/AKT pathway remains unknown, which specific mechanism needs to be further studied. Moreover, it has been found that the status of the pathway can influence the polarization of macrophages towards M2 subtype (Zhao et al., 2020), while the significance of M2 macrophages in asthma inflammation has not been fully elucidated (Saradna et al., 2018). Whether the downregulated pathway after BYD treatment can alleviate asthma airway inflammation or even airway remodeling by regulating M2 macrophage polarization remains unclear, nonetheless, it provides guidance for future research discovering the potential mechanism of BYD in the treatment of asthma. In our animal study, there was no intervention of BYD in the control group of normal mice, which is a limitation of our research. In the future, we will enrich the grouping and incorporate it into *in vitro* studies to further explore the mechanism of action of BYD.

## Conclusion

By integrating network pharmacology and molecular docking technology, this study has successfully identified the potential active components and molecular targets of BYD in asthma treatment. Moreover, the PI3K/AKT signaling pathway has been recognized as a crucial mechanism underlying the action of BYD. The results of animal study showed that the inhibitory effect of BYD on airway inflammation of asthma mice was potentially exerted through modulation of the AKTPI3K/AKT signaling pathway. These findings provide valuable insights into unraveling the mechanistic underpinnings of TCM in managing asthma using contemporary scientific approaches.

## Data Availability

The original contributions presented in the study are included in the article/supplementary materials, further inquiries can be directed to the corresponding author.
